# Establishment of lung cancer patient-derived xenograft models and primary cell lines for lung cancer study

**DOI:** 10.1186/s12967-018-1516-5

**Published:** 2018-05-22

**Authors:** Yanan Jiang, Jimin Zhao, Yi Zhang, Ke Li, Tiepeng Li, Xinhuan Chen, Simin Zhao, Song Zhao, Kangdong Liu, Ziming Dong

**Affiliations:** 1 Department of Pathophysiology, School of Basic Medical Sciences, Zhengzhou University, Zhengzhou, 450001 China; 2 Henan Provincial Cooperative Innovation Center for Cancer Chemoprevention, Zhengzhou, 450001 China; 3 The First Affiliated Hospital, Zhengzhou University, Zhengzhou, 450052 China; 4 The China-US (Henan) Hormel Cancer Institute, Zhengzhou, 450008 China; 5 The Affiliated Cancer Hospital, Zhengzhou University, Zhengzhou, 450008 China

**Keywords:** Lung cancer, Patient-derived xenograft, Signal pathway, Primary cell culture

## Abstract

**Background:**

The overall 5-year survival rate of lung cancer is about 15% even with therapeutic drugs like tyrosine kinase inhibitors. Ideal models are urgently needed for exploring mechanisms and finding new drugs. Patient-derived xenografts (PDX) models and primary cells are both used to screen therapeutic regimens for cancer. However, PDX models and primary cells from the same patient are difficult to establish. Their consistency to the original tumor tissue is not well studied.

**Methods:**

31 lung cancer patient tissues were procured to establish the lung cancer PDX models and primary cell lines. Tumor growth measurements, histological and immunohistochemistry analysis, Western blotting, *EGFR* and *K*-*RAS* mutation detection and gefitinib sensitive assay were performed to evaluate the characteristic of established PDX models. Immunofluorescence analysis, anchorage-independent cell growth, Western blotting and gefitinib sensitive assay were performed to assay the characteristic of established primary cell lines. The whole-exome sequencing was used to compare the characteristic of the patient’s tumor tissue, established PDX and primary cell line.

**Results:**

Twenty-one lung cancer PDX models (67.74%, 21/31) and ten primary cell lines (32.25%, 10/31) were established from patients’ tumor tissues. The histology and pathological immunohistochemistry of PDX xenografts are consistent with the patients’ tumor samples. Various signal pathways were activated in different PDX models (n = 5) and primary cell lines (n = 2). *EGFR* mutation PDX model and primary cell line (LG1) were sensitive to gefitinib treatment. The expression of CK8/18, TTF1 and NapsinA in LG1 and LG50 primary cells were also positive. And the activated signal pathways were activated in LG1 and LG50 primary cell lines. Furthermore, the gene mutation in PDX tumor tissues and primary cell line (LG50) was consistent with the mutation in LG50 patient’s tumor tissues.

**Conclusion:**

These data suggested that established lung cancer PDX models and primary cell lines reserved mostly molecular characteristics of primary lung cancer and could provide a new tool to further understand the mechanisms and explore new therapeutic strategies.

## Background

Lung cancer is the leading cause of cancer-related deaths worldwide [1–4]. Lung cancer treatments are dominated by chemotherapy, radiotherapy, surgery, even molecular targeted drugs [5, 6]. However, the overall 5-year survival rate of lung cancer is about 15% [7], even lower in small-cell lung carcinomas (SCLC) [5]. Molecular mechanism study of lung cancer is an immediate priority to find personal and targeted therapeutic strategies. In recent years, patient-derived xenografts have been established with the original molecular characteristics and heterogeneity of the cancer tissues [8–10]. These PDX models were even used to screen therapeutic regimens for breast cancer, gastric cancer and esophageal cancer [11–13]. Moreover, the primary cells are useful tools for mechanism study compared with stable cell lines, because of maintaining heterogeneity of tumors. The molecular changes in both PDX models and primary cell lines are valuable for mechanism research, new drug development and personalized treatments [14, 15].

Here, we established and characterized lung cancer patient-derived xenografts and established primary cell lines from patients’ tissues. We evaluated the pathological characteristic of the PDX models and primary cell lines. The differences between PDX models and the original tumors were also investigated. We found both the PDX models and primary cell lines mostly reserved molecular characteristics and heterogeneity of original cancer tissues, even drug sensitivity. Therefore, these PDX models and primary cell lines provide a platform for further understanding the molecular mechanisms and therapeutic screening, regimen for lung patient from cell to the animal level.

## Methods

### Patient tissue procurement

31 lung cancer patients had undergone the surgical procedures at the First Affiliated Hospital of Zhengzhou University (Zhengzhou, China) and lung cancer tissues were obtained from August 2014 to October 2015. All patients did not receive both chemotherapy and radiotherapy and followed written informed consent before surgery. All research protocols were approved by the research ethics committee of Zhengzhou University. Tissue histology was analyzed and confirmed by two pathologists. Lung cancer specimens were harvested from the periphery of whole tumor tissues to maintain high activity and low necrotic part. All tissues were transported to the lab in transport media (FBS-free PRMI DMEM with penicillin and streptomycin).

### Animals

6–8 weeks old female CB17/severe combined Immunodeficiency (SCID) mice of 18 ~ 20 g average body weight (Vital River, Beijing, China) were used in the studies. 4–5 mice were kept in a pathogen-free environment with light controlled rooms (12 h cycles) and provided with food and water adlibitum.

### Patient tumor xenografts

Tissue samples were placed in Petri dishes containing PBS with penicillin and streptomycin. The tissue was divided into four parts (implanted into SCID mice, fixed in 10% formalin, in liquid nitrogen for protein extraction and for primary cells). The detailed method of implanting into SCID mice was described in our previous works [12]. In brief, the tumor tissues were implanted subcutaneously in mice. The tumor was passaged when the tumor size reached ~ 1500 mm^3^. PDX models were used for study when the tumors were passaged for three generations. All animal studies were performed according to guidelines approved by the Zhengzhou University Institutional Animal Care and Use Committee.

### Tumor growth measurements

The tumors were measured with a vernier caliper twice a week. Tumor volume was calculated using the formula V = LD × (SD)^2^/2, where V was tumor volume, LD was longest tumor diameter, and SD was the shortest tumor diameter. Tumor growth curves were plotted as tumor volume.

### Histological and immunohistochemistry analysis

One part of tumor tissue was embedded into paraffin for histopathologic examination and immunohistochemistry analysis. All the slides were stained with Harris Hematoxylin after dewaxing 5 μm thick sections with dimethylbenzene and were evaluated by two pathologists. Tissue sections were incubated with CK5/6, P63, P40, or CK8/18, TTF1, NapsinA antibodies (Abcam, England) overnight at 4 °C. HRP-IgG secondary antibody was used at 37 °C for 15 min and detected by the diaminobenzidine (DAB) reactions. All slides were observed and measured by Olympus microscope (Japan).

### Western blotting

The PDX tissues were ground in liquid nitrogen and then were lysed. Next, protein was extracted by centrifugation for 30 min at 12,000 rpm. Protein (50 μg) was separated in 10% SDS-PAGE gel, and transferred to PVDF membrane at 90 V for 2 h. The membrane was blocked with 5% no-fat milk for 60 min. Later, membrane was incubated with mTOR, p-mTOR (Ser2481), STAT1, p-STAT1 (Tyr701), STAT3, p-STAT3 (Tyr705), AKT1, p-AKT (Ser473), ERK, p-ERK (Thr202/Tyr204) antibodies (Cell Signaling Technology, America) overnight at 4 °C. All antibodies were used at 1:1000. The STAT3 antibody was from mouse, other antibodies were from rabbit. The fluorescent secondary antibody was incubated at room temperature for 1.5 h. The PVDF membranes were scanned by Odyssey and analyzed by Image Studio Ver 2.1.

### *EGFR* and *K*-*RAS* mutation detection

Lung cancer patients’ tissues and xenograft tissues from PDX models were pathologically reviewed to ensure that tumor cells were more than 80% and that no significant tumor necrosis had occurred. Genomic DNA was extracted from each sample using Puregene Cell and Tissue Kit (QIAGEN, Cat#158388, Germany). The quantity and quality of DNA samples were measured by Nanodrop ND-1000 UV/VIS spectrophotometer (Thermo Scientific, USA). DNA fragment integrity was confirmed by electrophoresis using 1% agarose gel. The concentration of DNA samples was normalized to 20 ng/μl and stored at − 20 °C. ‘Hotspot’ mutations in epidermal growth factor receptor (*EGFR*) (exons 18, 19, 20, 21) and *K*-*RAS* (exons 2 and 3) were screened by the mutant-enriched liquid chip polymerase chain reaction method.

### Gefitinib treatment for PDX and primary cell lines

After establishing PDX models, we chose LG1 and LG50 PDX xenografts for gefitinib treatment. Mice were divided into two groups (10 mice per group) which were vehicle control and gefitinib treatment group. Once the tumor volumes reached approximately 25 mm^3^, mice were treated by oral gavages with vehicle control (0.9% NaCl) and gefitinib (100 mg/kg). Body weight and tumor size measurements were performed twice a week.

LG1 and LG50 primary cells (1 × 10^3^ per well) for proliferation were seeded into 96-well plates. After overnight incubation, cells were treated with different concentrations of geftinib (0, 0.25, 0.5 and 1 μM) and incubated for 24, 48, 72, or 96 h. CCK8 Solution (10 μl, Dojindo, Japan) was then added and cells were incubated for another 1.5–2 h. Absorbance was measured at 450 nm.

### Establishment of primary lung cancer cell lines from the patients’ lung cancer tissues

The fresh lung cancer tissues were minced into small pieces less than 1 mm^3^ using sterile eye scissors, followed by extensive washing in RPMI-DMEM medium and centrifuging at 300*g* for 5 min. Next, the tissues were resuspended in RPMI-DMEM medium containing collagenase II (Invitrogen, USA) at the concentration of 200 U/ml and digested for 2–4 h at 37 °C. The enzymatic digestion was stopped when most of the tissues became cell suspensions. Following washing in RPMI-DMEM and centrifuging at 300*g* for 5 min, cells were transferred into standard tissue culture coated flasks (Corning Life Sciences, USA) and cultured in the Defined Keratinocyte-Serum Free Medium (DK-SFM) supplemented with l-glutamine (Invitrogen, USA), EGF 20 ng/ml, basic-FGF 10 ng/ml (PeproTech Inc., USA), 2% B27 (Invitrogen, USA), penicillin and streptomycin, and amphotericin B (0.25 mg/ml; Invitrogen, USA). All primary cells were cultured at 37 °C in a humidified incubator with 5% CO_2_. Culture medium was changed every 2–3 days. Cells were passaged after 80–90% confluence.

### Immunofluorescence analysis

2.5 × 10^5^ primary cells were seeded in 24-well plate which was placed a sterilized glass slide in every well, incubated for 24 h, and fixed with 4% paraformaldehyde for 30 min. CK8/18, TTF1, NapsinA antibodies (Rat anti-human, 1:50; Santa Cruz Biotechnology) was incubated at 4 °C overnight, and then FITC-conjugated rabbit IgG antibody was used. Cells with green fluorescent signals in the nucleus and cytoplasm were counted as positive expression cells. The In Cell 6000 Analyzer (GE) was used for detecting the fluorescence.

### Anchorage-independent cell growth

A total of 8000 primary cells were suspended in 0.33% basal medium eagle agar supplemented with 10% FBS. The cells were maintained at 37 °C, 5% CO_2_ for 2–3 weeks, and cell colony numbers were counted by In Cell 6000 Analyzer (GE).

### Whole-exome sequencing

We used the Illumina HiSeq to perform whole-exome sequencing of LG50 patient tissue, LG50 PDX xenografts, and LG50 primary cell line. All genomic variations including SNPs and In-Dels were detected by the software, such as HaplotypeCaller of GATK (v3.3.0). After that, the hard-filtering method was applied to get high-confident variant calls. Then the SnpEff tool (http://snpeff.sourceforge.net/SnpEff_manual.html) was applied to perform a series of annotations for variants. The final variants and annotation results were used in the advanced downstream analysis.

### Statistical analysis

All statistical analyses were performed using SPSS statistical software 17.0. Experimental values were reported as mean ± standard deviation. One-way analysis of variance was used for statistical analysis. *p *< 0.05 was considered as statistically significant.

## Results

### Clinical characteristics of the patients

In this study, 31 lung cancer patients underwent surgical resection. These patients consisted of twenty-six men and five women ranging from 40 to 75 years in age (60.5 ± 10.0 years). All the patients did not have any apparent distant metastases before surgery and had not been previously treated. In these samples, there are 10 adenocarcinoma samples, 1 adenosquamous carcinoma, 1 mucoepidermoid carcinoma, 1 neuroendocrine and SCLC, 1 non-small cell lung carcinomas (NSCLC), 1 SCLC, 16 squamous carcinomas. There are 2 well-differentiated samples, 4 moderately-well, 12 moderate, 7 moderately-poor and 6 poor differentiated samples. According to lymph node metastasis, 8 of 11 samples with lymph node metastasis had been established, and 13 of 20 samples without lymph node metastasis had been established. All patients’ clinical data were shown in Table 1.

**Table 1:** Summary of the clinical data of lung cancer patients for PDX models

#	Number	Age	Sex	Pattern of organization	Tumor differentiation	TNM Staging	Lymph Node Metastasis	Engrafted model	Primary cell lines	*EGFR* mutation
1	LG1	45	Female	Adenocarcinoma	Poor	T2bN0M0	Y	Y	Y	L858R
2	LG2	69	Female	Adenocarcinoma	Moderate	T1aN0M0	N	Y	N	Exon19 delete
3	LG3	74	Male	Adenocarcinoma	Moderately-poor	T1bN0M0	N	Y	N	Wild type
4	LG6	60	Male	Squamous carcinoma	Moderately-poor	T2aN0M0	N	Y	Y	Wild type
5	LG7	43	Male	Mucoepidermoid carcinoma	Moderately-poor	T2bN0M0	Y	Y	Y	Wild type
6	LG10	62	Female	Adenocarcinoma	Moderate	T1N0M0	N	N	N	Wild type
7	LG12	75	Male	Squamous carcinoma	Moderately-poor	T2bN0M0	N	Y	Y	Wild type
8	LG14	77	Male	NE and SCLC	Well	T2N1M0	Y	Y	N	Wild type
9	LG17	72	Male	Adenocarcinoma	Moderately-good	T2N0M0	N	Y	N	Wild type
10	LG19	53	Male	Squamous carcinoma	Moderate	T1N0M0	N	N	Y	Wild type
11	LG21	67	Male	Adenocarcinoma	Moderate	T2N0M0	N	N	N	Wild type
12	LG22	55	Male	Squamous carcinoma	Moderate	T2aN0M0	N	Y	N	L858R
13	LG24	40	Male	SCLC	Moderately-poor	T2N0M0	N	Y	N	Wild type
14	LG26	50	Male	Squamous carcinoma	Moderately-poor	T2bN0M0	N	Y	N	Wild type *K*-*RAS*
15	LG28	60	Male	Squamous carcinoma	Moderate	T2bN1M0	Y	Y	N	colon12 GGT > CGT
16	LG29	58	Male	Squamous carcinoma	Moderately-good	T2bN0M0	N	Y	N	Wild type
17	LG33	58	Male	Squamous carcinoma	Moderate	T3N1M0	N	Y	N	Wild type
18	LG37	64	Male	Squamous carcinoma	Poor	T4N1M0	Y	Y	Y	Wild type
19	LG38	46	Male	Squamous carcinoma	Moderately-good	T2N0M0	N	Y	N	Wild type
20	LG42	67	Male	Squamous carcinoma	Moderately-good	T2N1M0	Y	N	N	Wild type
21	LG44	58	Male	NSCLC	Poor	T2aN0M0	N	Y	N	Wild type
22	LG46	56	Female	Squamous carcinoma	Poor	T2aN1M0	Y	Y	N	Wild type
23	LG48	50	Male	Adenocarcinoma	Moderate	T2N1M0	Y	N	Y	Wild type
24	LG50	75	Female	Adenocarcinoma	Moderate	T2N0M0	N	Y	Y	Wild type
25	LG52	56	Male	Squamous carcinoma	Moderate	T1N1M0	Y	N	N	Wild type
26	LG53	65	Male	Adenosquamous carcinoma	Poor	T2aN1M0	Y	Y	N	Wild type
27	LG54	70	Male	Adenocarcinoma	Moderate	T1N0M0	N	N	N	Wild type
28	LG55	64	Male	Adenocarcinoma	Moderate	T2aN0M0	N	N	Y	Wild type
29	LG59	68	Male	Squamous carcinoma	Well	T1N0M0	N	N	N	Wild type
30	LG61	59	Male	Squamous carcinoma	Moderately-poor	T2N2M0	Y	Y	Y	Wild type
31	LG62	67	Male	Adenocarcinoma	Poor	T2bN0M0	N	N	N	Wild type

*Y* yes; *N* no

### Growth of lung cancer xenografts in SCID mice

**Fig. 1:**
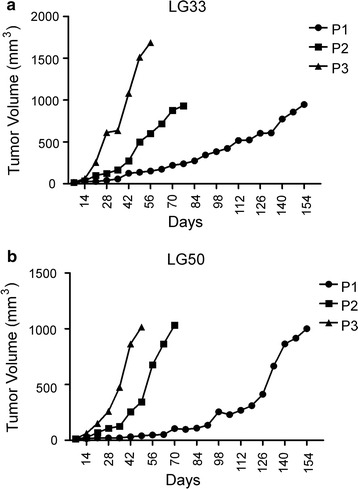
Growth curves of lung cancer PDXs in SCID mice. **a** The generation harboring the patient-derived tumor tissue was termed P0, with subsequent generations numbered consecutively (P1, P2, P3 and so on). The growth of PDX xenografts reaches a steady state level in third and more passages. Growth curves of three passage xenografts (1st, 2nd and 3rd passage) plotted as tumor volume in PDX xnografts LG33. **b** The tumor volume of 1st, 2nd and 3rd passage was measured in lung adenocarcinoma LG50

31 tumor specimens were transplanted to SCID mice and 21 xenografts were established (Table 1). The successful growth rate is 67.7%. The pathological types included SCLC, adenocarcinomas and squamous carcinomas. The generation harboring the patient-derived tumor tissue was termed P0, with subsequent generations numbered consecutively (P1, P2, P3 and so on). The growth curves of three passages (P1, P2, and P3) of LG50 and LG33 (patient ID) were plotted using the data obtained each week (Fig. 1). The latency time before passaging of P1 for LG50 and LG33 was 168 and 154 days, respectively, the latency time of P2 was 70 and 77 days, and P3 was 35 and 56 days. It was noticed that the latency time of each passage became shorter and it decreased to 1–3 months in subsequent passages after P3.

### Histology and immunohistochemistry comparison between xenografts and patients’ tumors

**Fig. 2:**
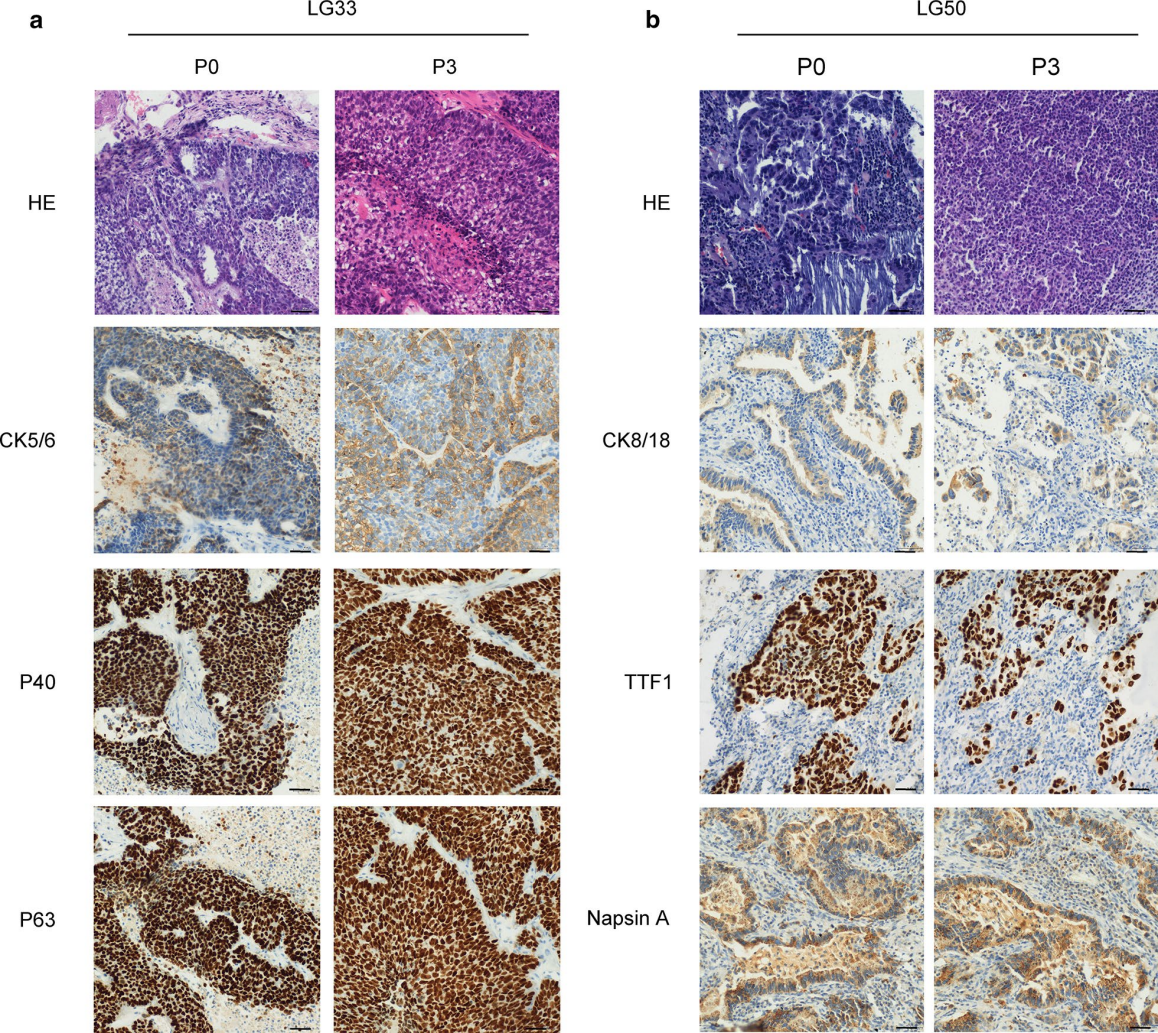
Histologic and immunohistochemistric features of patients’ lung cancers and their corresponding xenograft. **a** Histologic sections (H&E) and CK5/6, p40, p63 expression in LG33 lung cancer patient’s tissues and the third passage xenografts (Bar: 50 μm). Tumor tissue slides from each group were prepared with paraffin sections after fixation with formalin and then stained with HE and the indicated antibodies. P40, P63 and CK5/6 were specific clinical diagnosis indexes for squamous carcinoma. Expression of CK5/6, p40 and p63 were visualized by microscopy in LG33. **b** Histologic sections (H&E) and CK8/18, TTF1, NapsinA expression in LG50 lung cancer patient’s tissues and the third passage xenografts (Bar: 50 μm). CK8/18, TTF1 and NapsinA were specific clinical diagnosis indexes for adenocarcinoma. Expression of CK8/18, TTF1 and NapsinA were visualized by microscopy in LG50

Next, LG33 with squamous carcinoma and LG50 with adenocarcinoma were used as representative to compare histology and immunohistochemistry of the primary tumor tissue with P3 PDX tumor tissues. The HE staining results indicated that the morphology was similar between primary tumor tissues and PDX tumor tissues (Fig. 2a, b). P40, P63 and CK5/6 were specific clinical diagnosis indexes for squamous carcinoma. CK8/18, TTF1 and NapsinA were specific clinical diagnosis indexes for adenocarcinoma. Our results indicated CK5/6, p40, and p63 were positive in both P0 and P3 of lung squamous carcinoma LG33 (Fig. 2a) and CK8/18, NapsinA, and TTF-1 were positive in both P0 and P3 of lung adenocarcinoma LG50 (Fig. 2b). These results indicated that the original tumors’ characteristics were maintained in established PDX models.

### Activated signal transduction pathways in the established lung cancer PDXs

**Fig. 3:**
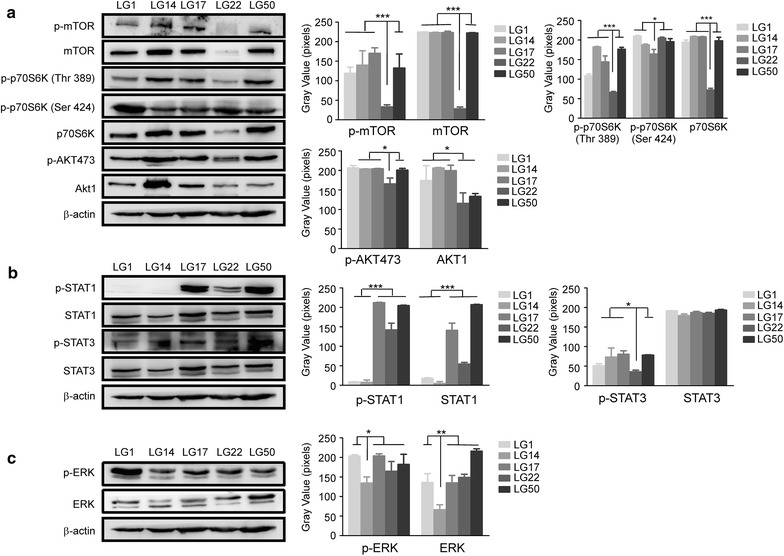
Activated signal transduction pathways in the established lung cancer PDXs. **a** AKT-mTOR axis pathway was activated in the different PDXs (LG1, LG14, LG17, LG22 and LG50). Protein levels of p-mTOR and mTOR, p-p70S6K (Thr389), p-p70S6K (Ser424) and p70S6K, p-AKT473 and AKT1 were visualized by Western blotting. β-actin was used to verify equal protein loading. **b** STAT1 and STAT3 pathway were activated in the different PDXs (LG1, LG14, LG17, LG22 and LG50). Protein levels of p-STAT1 and STAT1, p-STAT3 and STAT3 were visualized by Western blotting. β-actin was used to verify equal protein loading. **c** ERK pathway were activated in the different PDXs (LG1, LG14, LG17, LG22 and LG50). Protein levels of p-ERK and ERK was visualized by Western blotting. β-actin was used to verify equal protein loading. Each experiment was repeated three times. According to the results of Western blotting, we used image J to determine the gray value of each stripe, made histograms by the gray value, the asterisks (*,**, ***) indicate a significant (*p *< 0.05, 0.01, *p *< 0.001, respectively)

mTOR, p70S6K, Akt, STAT1, STAT3, and ERK, as well as their corresponding phosphorylated forms, were examined in PDX models by Western blotting. LG1, LG14, LG17, LG22 and LG50 were representative as PDX models. Among these models, the level of p-mTOR, p-AKT473 and p-p70S6K (Thr389) was lowest in LG22. p-p70S6K (Ser424) in LG17 (Fig. 3a). p-STAT1 was lowest in LG1 and LG14. However, p-STAT3 was lower in LG1 and LG22 (Fig. 3b). Moreover, p-ERK was lowest in LG14 (Fig. 3c).

### *EGFR* and *K*-*Ras* mutation and gefitinib sensitive assay

**Fig. 4:**
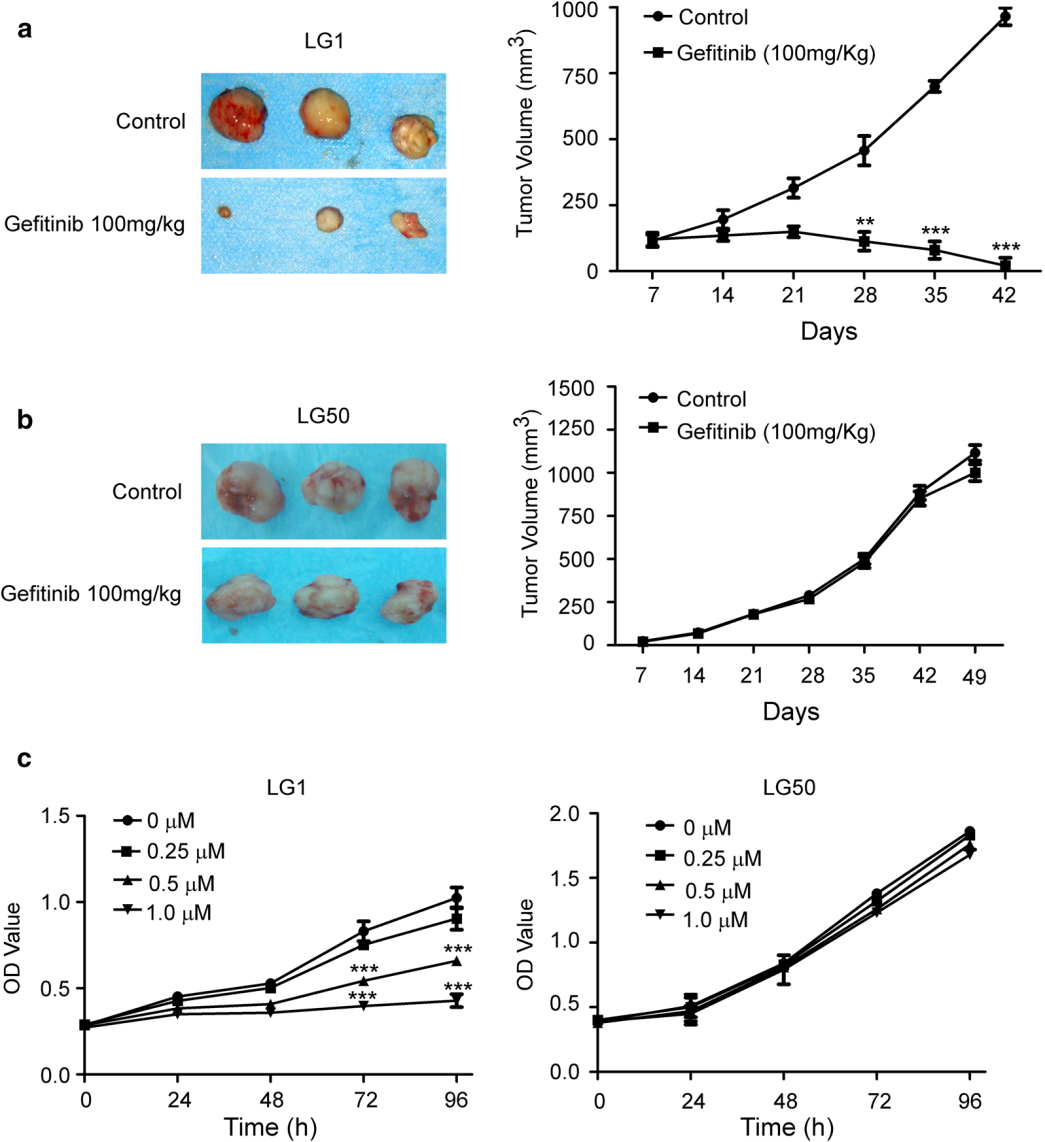
Gefitinib inhibits tumor cell growth in *EGFR* mutant PDX model and its corresponding primary cell. **a** Gefitinib (100 mg/kg) significantly suppresses tumor growth in an EGFR mutant PDX LG1. Mice were divided into two different groups as described in Materials and Methods. The asterisks (**, ***) indicate a significant (*p *< 0.01, *p *< 0.001, respectively). **b** Gefitinib (100 mg/kg) have no effective on tumor growth in *EGFR* wild type PDX LG50. Mice were divided into two different groups as described in Materials and Methods. There is no statistical significance in tumor volume compared to the vehicle-treated control (*p *> 0.05). **c** Gefitnib suppresses LG1 primary cell proliferation and have no effection on the LG50 primary cell proliferation. LG1 primary cells and LG50 primary cells were treated with different doses of gefitinib (0, 0.25, 0.5 and 1 μM), and proliferation was measured by CCK8 assay Significant differences were evaluated using a 2-way ANOVA to determine significant time and dose effects. The asterisks (**, ***) indicate a significant (*p *< 0.01, *p* < 0.001, respectively). There was no significant differences in the LG50 cell proliferation. The morphological characteristic was obseved by microscopes (Bar:100 μm)

We sequenced *EGFR* and *K*-*Ras* gene locus of established PDX models. We found 2 *EGFR* L858R mutation, 1 *EGFR* Exon19 deletion mutation and 1 *K*-*Ras* codon12 GGT > CGT mutation (Table 1). Next, we chose LG1 xenografts with *EGFR* L858R and LG50 xenografts with wild-type *EGFR* to do the gefitinib treatment sensitive assay. We found that LG1 xenografts quickly shrunk after injecting gefitinib 100 mg/kg once daily for a week. By comparison, LG50 grew normally. This indicated LG1 was sensitive to gefitinib treatment (Fig. 4a), and LG50 was resistant (Fig. 4b). To assess the gefitinib sensitivity, LG1 primary cells and LG50 primary cells were treated by different doses of gefitinib (0, 0.25, 0.5 and 1 μM). Our results found that gefitinib suppressed the LG1 cell proliferation and had no effection on the LG50 primary cell proliferation by CCK8 assay (Fig. 4c). These indicated that EGFR mutant model and primary cells were sensitive to gefitinib.

### Immunofluorescence analysis of primary cells

**Fig. 5:**
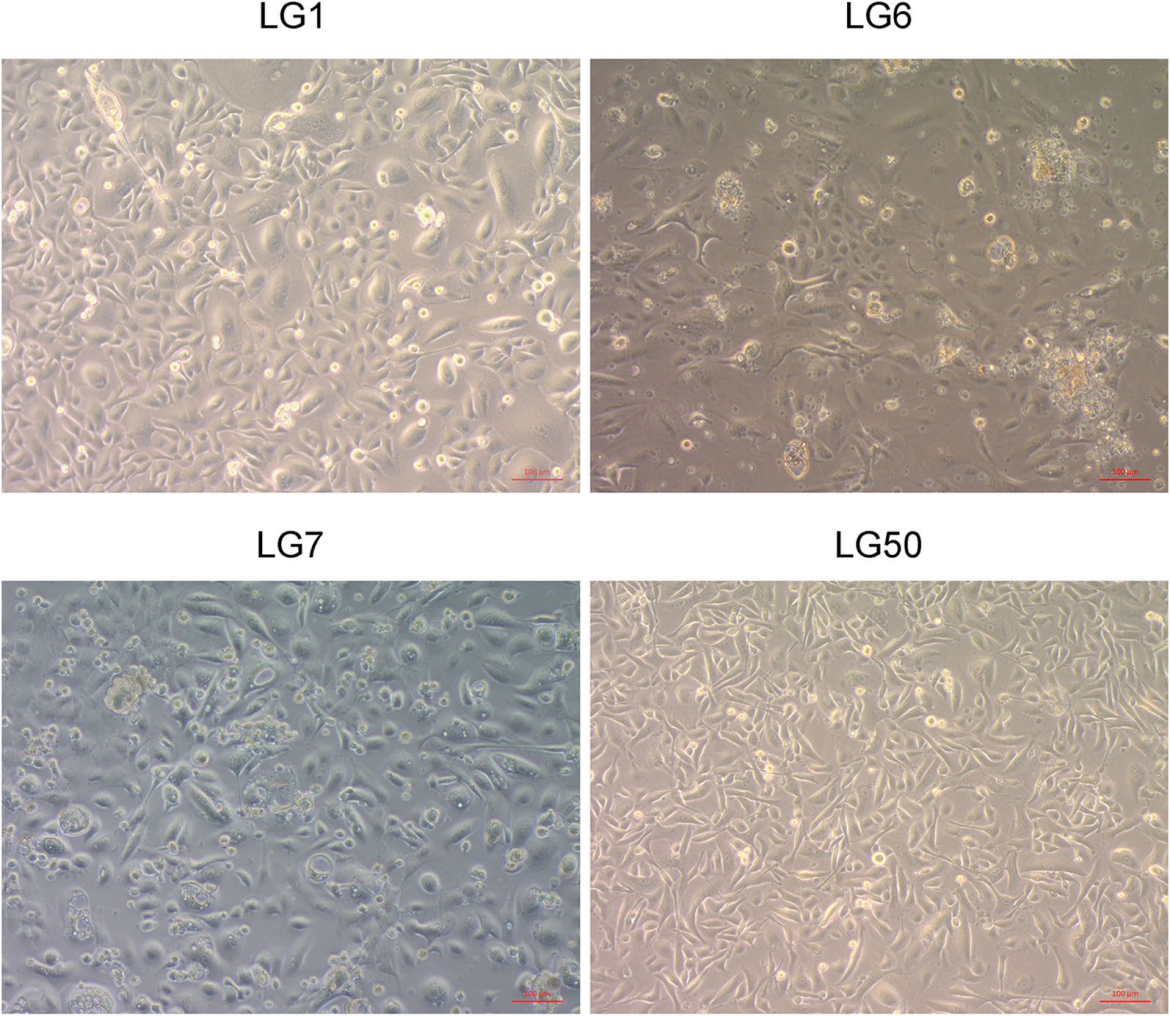
Morphological characteristic of LG1, LG6, LG7 and LG50 lung cancer primary cells. Primary cell line was established from the fresh lung cancer tissues LG1, LG6. LG7 and LG50

**Fig. 6:**
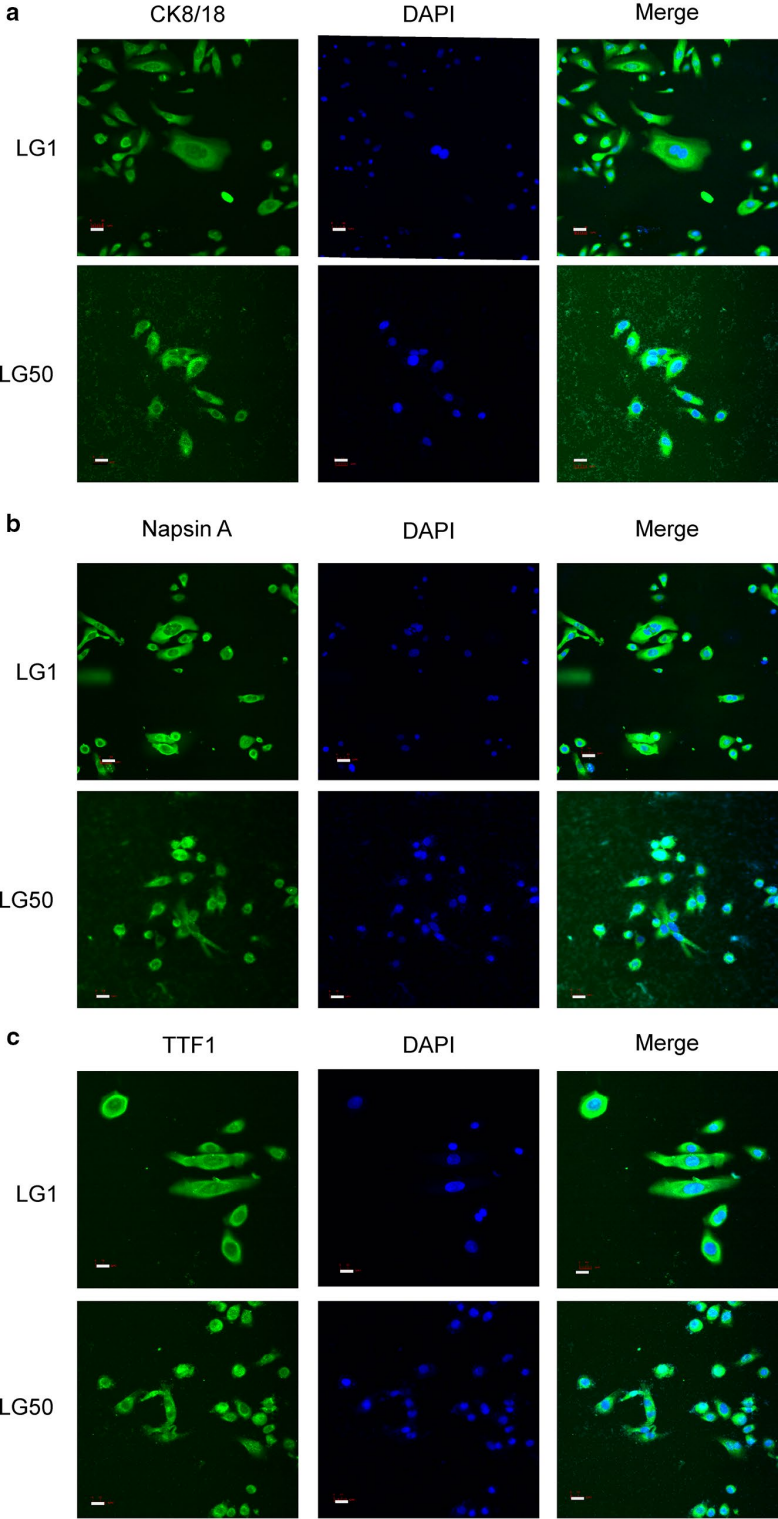
Immunofluorescence analysis of primary cells in LG1 and LG50. **a** CK8/18 was positive in LG1 and LG50 primary cells. LG1 and LG50 primary cells were fixed and subjected to immunofluorescence analysis. Sections were stained as described in “Methods” section using anti-CK8/18 followed by FITC-conjugated anti-rabbit IgG. Nuclei were counterstained with DAPI (Bar:15 μm). **b** Napsin A was positive in LG1 and LG50 primary cells. LG1 and LG50 primary cells were fixed and subjected to immunofluorescence analysis. Sections were stained as described in “Methods” section using anti-NapsinA followed by FITC-conjugated anti-rabbit IgG. Nuclei were counterstained with DAPI (Bar:15 μm). **c** TTF1 was positive in LG1 and LG50 primary cells. LG1 and LG50 primary cells were fixed and subjected to immunofluorescence analysis. Sections were stained as described in “Methods” section using anti-TTF1 followed by FITC-conjugated anti-rabbit IgG. Nuclei were counterstained with DAPI (Bar:15 μm)

10 primary cell lines were successfully established. LG1, LG6, LG7 and LG50 was respective as these primary cell lines. The primary cells had the cellular atypia and pleomorphism (Fig. 5). Next, we evulate whether the cells have the primary characteristic of tumor cells. LG1 and LG50 as adenocarcinoma were tested by the clinical indexes (CK8/18, NapsinA, and TTF1). Immunofluorescence assay indicated the expresion of these indexes were positive in both LG1 and LG50 primary cells (Fig. 6).

### Anchorage-independent growth and activated signal transduction pathway in established primary cells

**Fig. 7:**
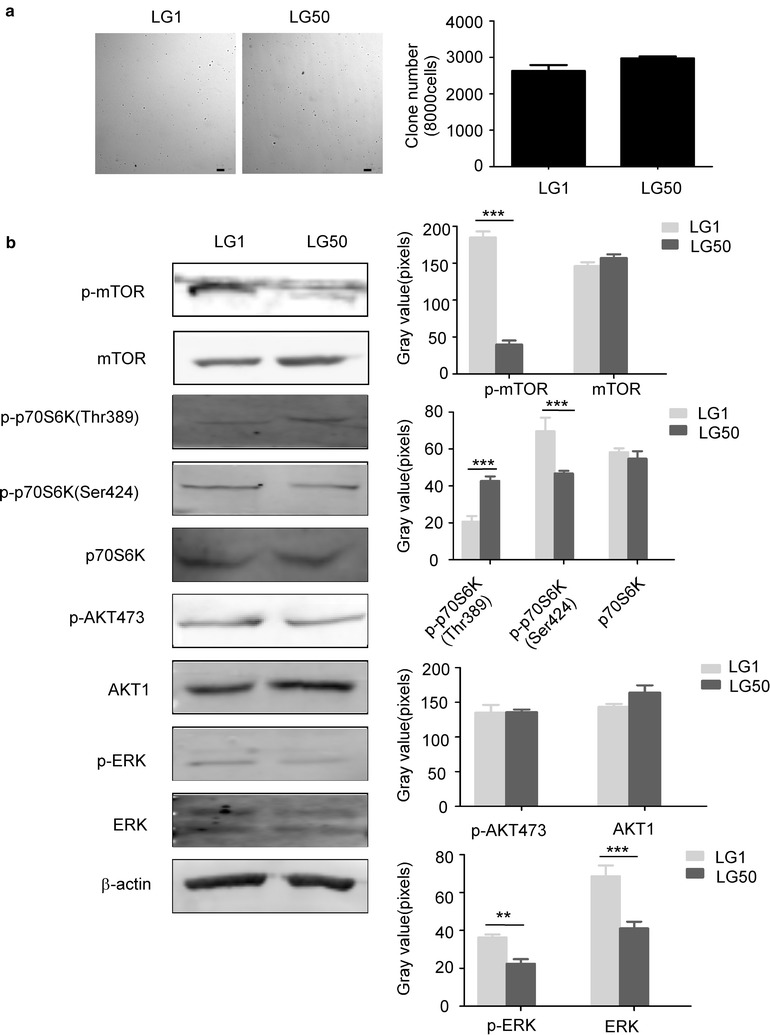
Anchorage-independent growth assay and activated signal transduction pathways in LG1 and LG50 primary cells. **a** Representative images from the anchorage-independent growth assay for LG1 (left) and LG50 (right) (Bar:100 μm). Data are presented as mean values ± S.D. from triplicate experiments. **b** Activated signal transduction pathways in LG1 and LG50 primary cells. The levels of phosphorylated and total proteins of AKT-mTOR axis, ERK in LG1 and LG50 primary cells were visualized by Western blotting. β-actin was used to verify equal protein loading. Each experiment was repeated three times. According to the results of Western blotting, we used image J to determine the gray value of each stripe, made histograms by the gray value, and analyzed by SPSS 17.0. The asterisks (**, ***) indicate a significant (*p *< 0.01, *p* < 0.001, respectively)

We evaluated the characteristics of these primary tumor cells by anchorage-independent growth assay and found LG1 and LG50 primary cells both grow independently (Fig. 7a). The clone number of LG1 and LG50 was 2632.81 ± 275.89 and 2794.2 ± 93.54 every 8000 cells, respectively. The expressions of mTOR, p70S6K, AKT, and ERK, as well as their phosphorylation forms, were examined in both LG1 and LG50 primary cells. The level of p-mTOR, p-AKT473, and p-p70S6K (Thr389) was different between the LG1 and LG50 primary cell lines (Fig. 7b). These indicated different signal pathways were activated in different lung cancer; even they had the same pathology.

### Whole-exome sequencing

**Fig. 8:**
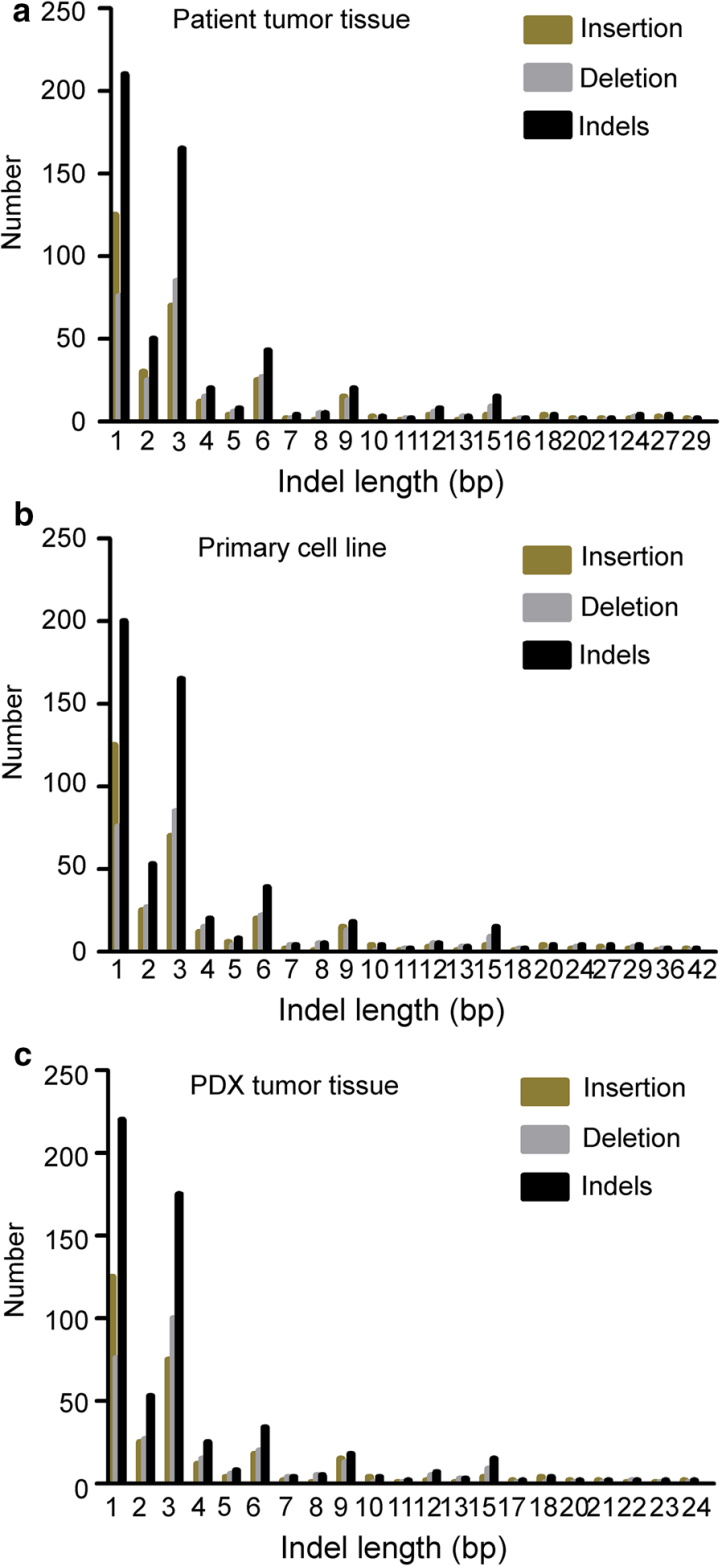
The distribution of lengths of coding InDel variants (CDS) in LG50 patient tumor tissue, primary cell line and PDX tumor tissue. **a** The distribution of lengths of CDS was in LG50 patient tumor tissue. DNA was extracted from LG50 patient tumor tissue and was performed the whole-exome sequencing by Illumina HiSeq. There were InDels in this sample. The length distribution of the InDels, Deletion and Insertion in CDS were also plotted. **b** The distribution of lengths of CDS was in LG50 primary cells. DNA was extracted from LG50 primary cell line and was performed whole-exome sequencing by Illumina HiSeq. There were InDels in this sample. The length distribution of the InDels, Deletion and Insertion in CDS were also plotted. **c** The distribution of CDS was in LG50 PDX tumor tissues. DNA was extracted from LG50 PDX tumor tissue and was performed whole-exome sequencing by Illumina HiSeq. There were InDels in this sample. The length distribution of the InDels, deletion and insertion in CDS were also plotted

To evaluate the genetic variations between the patient’s tumors and the established models, single nucleotide polymorphisms (SNPs) and INsertion/DELetion (InDel) were analyzed among LG50 patient tissue, LG50 primary cells, and PDX tumor tissue. The summary statistics of SNPs was shown in Table 2. There were 102,402, 94,847, 95,908 SNPs in the patient tissue, primary cell, and PDX tissue respectively. 98.23, 98.31, 94.59% were represented in dbSNP, while 95.81, 95.81, 92.17% were annotated in the 1000 genomes project database, respectively. In these genes, there are similar synonymous, missense, stop loss, stop-gain, start loss in three samples in Table 3. Moreover, the summary statistics of InDels was shown in Table 4. There were 15,964, 14,176, 14,958 InDels in three samples, respectively. In these variants, 80.62, 82.15, 78% were represented in dbSNP and 60.42, 60.77, 57.28% were annotated in the 1000 genomes project database. The number of novel InDels was 2728, 2223 and 2955, respectively. In three InDels groups in Table 5, 250, 252, 264 were frameshift, 7, 7, 9 were stop loss, 2 were all start loss and 59, 55, 58 were splice site. The length distribution of the InDels in coding sequence region (CDS) were also plotted in Fig. 8.

**Table 2:** Summary statistics for identified SNPs for LG50 tumor tissue, primary cell line and PDX xenografts

Samples	Total SNPs	Fraction of SNPs in dbSNP (%)	Fraction of SNPs in 1000 genomes (%)	Novel	Homozygous	Heterozygous	Intron	5′ UTRs	3′ UTRs	Upstream	Down stream	Intergenic	Ti/Tv
Patient tumor tissue	102,402	98.23	95.81	1473	1473	54,009	69,062	1567	3298	2077	1538	2330	2.34
Primary cell line	94,847	98.23	95.81	1473	44,541	50,306	62,661	1426	2974	1793	1377	2188	2.35
PDX tumor tissue	95,908	94.59	92.17	1473	44,875	51,033	61,376	1468	3130	1778	1359	2170	2.33

**Table 3:** Functional categories for coding SNPs for LG50 tumor tissue, primary cell line and PDX xenografts

Samples	Synonymous	Missense	Stopgain	Stoploss	Startloss	Splicing
Patient tumor tissue	10,587	9794	79	33	16	80
Primary cell line	10,536	9774	80	32	17	79
PDX tumor tissue	12,431	10,036	82	31	15	89

**Table 4:** Summary statistics for identified InDels for LG50 tumor tissue, primary cell line and PDX xenografts

Samples	Total InDels	Fraction of InDels in dbSNP (%)	Fraction of InDels in 1000 genomes (%)	Novel	Homozygous	Heterozygous	Intron	5′ UTRs	3′ UTRs	Upstream	Down stream	Intergenic
Patient tumor tissue	15,964	80.62	60.42	2728	6044	9920	13,059	234	664	340	296	339
Primary cell line	14,176	82.15	60.77	2223	5567	8609	11,616	195	561	264	242	309
PDX tumor tissue	14,958	78.09	57.28	2223	5379	9579	12,205	214	616	273	259	328

**Table 5:** Functional categories for coding InDels for LG50 tumor tissue, primary cell line and PDX xenografts

Samples	Frameshift	Non-frameshift insertion	Non-frameshift deletion	Stoploss	Startloss	Splicing
Patient tumor tissue	250	122	142	7	2	59
Primary cell line	252	111	144	9	2	55
PDX tumor tissue	264	117	144	9	2	58

## Discussion

Identification and characterization of tumor molecular changes and genetics are pivotal for screening targeted drugs and precise treatments [8, 16, 17]. PDXs maintain the histology, as well as the molecular and genetic characteristics of the original tumor. Therefore, PDX models have advantages over conventional cell-line-derived xenografts and other models [10]. Here, we established 21 lung cancer PDX models from 31 lung cancer patients, and the success rate is 67.7% which is higher than others [8, 18]. Monitoring of tumor growth and location was important for evaluating the PDX models [19]. One defining feature of PDX model is its lag time before exponential growth that can be stable or variable during subsequent passages of the tumor. PDX tumor reached a steady state level in third and more passages, probably due to the progressive substitution of the original human tumor stroma with SCID mice stroma. In our PDX models, the first passage reached 1500 mm^3^ on day 168 and 154 for LG50 and LG33, respectively.

Different pathological type of cancer have different indexes in clinical. P40, P63 and CK5/6 were specific clinical diagnosis indexes for squamous carcinoma. CK8/18, TTF1 and NapsinA were specific clinical diagnosis indexes for adenocarcinoma. Our results found lung squamous carcinoma indexes and lung adenocarcinoma indexes still maintained in the third passage of the PDX models. These data indicated that our PDX models reflected the characteristics of patients’ samples. Different signaling pathways such as AKT/mTOR pathway, STAT1, and STAT3 pathway, ERK pathway played a critical role in the progression of lung cancer [20–22]. Targeting mTOR and AKT is a promising way to personalized treatment of lung cancer [23, 24]. AKT/mTOR pathways were strongly activated in established PDX models and primary cell lines (Figs. 3 and 7). Even in the same pathological type of lung cancer, the different pathways were activated. These indicated that our models provided a platform to screen the individual pathway inhibitors in the future.

Genetic and epigenetic abnormalities of primary cancer influence the processes of invasion, metastasis and drug resistance [6, 25, 26]. *EGFR* plays an essential role in regulating cell proliferation and apoptosis of lung adenocarcinoma. *EGFR* mutation and *K*-*Ras* mutation are regarded as mutation initiator in lung cancer patients [27, 28]. Mutation of *EGFR* in 17% of NSCLC patients is more frequent than in 5% of SCLC [20]. In the established PDX models, we found 4 *EGFR* mutation and 1 *K*-*Ras* mutation. We assessed the EGFR inhibitor sensitivity of these models. PDX model with *EGFR* L858R mutation was sensitive to gefitinib. However, PDX model with *EGFR* wild-type was resistant to gefitinib (Fig. 5). These results indicated PDX models are suitable tools for studying molecular diversity, drug screening and precise therapies.

The primary cell lines have advantages over conventional cell lines which may lose the diversity of tumor cells. Therefore, we established the primary cell lines from the patients’ lung cancer tissues. We checked the molecular characteristics of these primary cell lines. CK8/18, TTF1, and NapsinA were mostly located in the cytoplasm and cell nucleus of the LG1 and LG50 primary cells (Fig. 6). The LG1 and LG50 primary cells have a colony formation by anchorage-independent growth assay. Activated pathways in primary cells were also different (Fig. 7). It shows that primary cell lines maintain the tumor’s heteromorphism. Whole-exome sequencing is an important method to understand the diversity of tumor [29, 30]. Whole-exome sequencing results found that the established primary cell line and the PDX model remained more than 90% gene characteristics of the patient’s tumor tissues in LG50 (Tables 2, 3, 4, 5 and Fig. 8). Even through we indicated the established primary cell line and the PDX model remained more than 90% gene characteristics of the patient’s tumor tissues, more samples are needed to verify this result. We will continue amplify the library and put into clinical test.

## Conclusions

We established lung cancer PDX models and paired primary cell lines. The established PDX models and primary cell lines have uttermostly reserved molecular characteristics, heterogeneity, and drug sensitivity of patients’ tumor. These PDX models and primary cell lines provide a useful tool for understanding the molecular mechanisms and screening new compounds for lung cancer therapy.

## Abbreviations

TKIs: tyrosine kinase inhibitors
PDX: patient-derived xenografts
SCLC: small-cell lung carcinomas
SCID: severe combined immunodeficiency
NSCLC: non-small cell lung carcinomas
SNPs: single nucleotide polymorphisms
InDel: INsertion/DELetion
CDS: coding sequence region
